# Selenoproteins: Zoom-In to Their Metal-Binding Properties in Neurodegenerative Diseases

**DOI:** 10.3390/ijms26031305

**Published:** 2025-02-03

**Authors:** Carmen Duță, Corina Muscurel, Carmen Beatrice Dogaru, Irina Stoian

**Affiliations:** Department of Biochemistry, Carol Davila University of Medicine and Pharmacy, 050474 Bucharest, Romania; carmen.duta@umfcd.ro (C.D.); corina.muscurel@umfcd.ro (C.M.); irina.stoian@umfcd.ro (I.S.)

**Keywords:** selenoproteins, SELENOP, transition metals, Alzheimer’s disease, Parkinson’s disease

## Abstract

Selenoproteins contain selenium (Se), which is included in the 21st proteinogenic amino acid selenocysteine (Sec). Selenium (Se) is an essential trace element that exerts its biological actions mainly through selenoproteins. Selenoproteins have crucial roles in maintaining healthy brain activity. At the same time, brain-function-associated selenoproteins may also be involved in neurodegenerative diseases, such as Alzheimer’s disease (AD) and Parkinson’s disease (PD). The selenoproteins GPx4 (glutathione peroxidase 4), GPx1 (glutathione peroxidase 1), SELENOP (selenoprotein P), SELENOK (selenoprotein K), SELENOS (selenoprotein S), SELENOW (selenoprotein W), and SELENOT (selenoprotein T) are highly expressed, specifically in AD-related brain regions being closely correlated to brain function. Only a few selenoproteins, mentioned above (especially SELENOP), can bind transition and heavy metals. Metal ion homeostasis accomplishes the vital physiological function of the brain. Dyshomeostasis of these metals induces and entertains neurodegenerative diseases. In this review, we described some of the proposed and established mechanisms underlying the actions and properties of the above-mentioned selenoproteins having the characteristic feature of binding transition or heavy metals.

## 1. Introduction

Selenium (Se) is a vital trace element that plays essential roles in many different biochemical processes of the organism. Se primarily functions in the body being incorporated into selenoproteins (approximately 80%) [1], which are involved in various conditions, such as oxidative stress (including endoplasmic reticulum stress), immunity, cancer, and inflammation.

According to the 2002 FAO/WHO dietary recommendations report, the intake of Se should include 26 μg/day for women and 34 μg/day for men. At a dose of 150–200 μg/day, Se has antioxidant, immune-enhancing, and anticarcinogenic effects.

Se deficiency is characterized by poor appetite, retarded body growth, and reduced total muscle mass. Two endemic diseases are directly associated with severe selenium deficiency caused by poor soil selenium content in regions of China (Tibet) and Russia: Kashin-Beck and Keshan. Kashin-Beck disease is an osteoarthritis (osteochondropathy) characterized by the atrophy, degeneration, and necrosis of cartilage tissue, followed by enlarged joints, shortened fingers and toes, and dwarfism in extreme cases. Keshan disease is a muscular disorder characterized by cardiomyopathy. Selenium deficiency also affects thyroid functions, cardiovascular and immune systems, and may cause infertility. In addition, the pathological effects are caused by the decrease in the selenoprotein synthesis [1,2].

Acute selenium excess (acute selenosis) symptoms appear in intoxication with an extremely high dose of dietary selenium ingested within a short period and may cause death. Chronic selenosis is produced by the ingestion of moderately elevated amounts of Se within a longer period. It is characterized by garlic breath, body mass loss, hair loss, changes in nail structure, dermatitis, gastrointestinal tract disorders, abnormal nervous system function (numbness, dysesthesia—“pins and needles”, hyper-reflexia, convulsions, motor weakness, paralysis, and occasional hemiplegia), the disruption of thyroid and growth hormone synthesis and insulin-like growth factor metabolism, hepatotoxicity, the impairment of natural killer cells, reduced fertility, and abnormalities in offspring. In medical practice, selenosis is quite rare; 80% of the population is diagnosed with a dietary deficiency of Se rather than its excess, and if selenosis is diagnosed, then it can be reversed solely by correcting the diet [1,3,4].

Selenoproteins are important in maintaining healthy brain function. Several selenoproteins are characterized by the property of binding metals both in physiological and pathological states. In the brain, selenoproteins can bind transition metal ions, such as iron, zinc, and copper, to sustain neural cell processes. SELENOP has a central role in binding metal ions. Normal brain activity also depends on maintaining the homeostasis of metal ions. Dyshomeostasis induces brain disorders. In addition, in the pathological states, selenoproteins are highly expressed in AD-related brain regions. It is well known that metal ions are involved, besides selenoproteins, in neurodegenerative diseases like AD and PD.

## 2. Metal-Binding Properties of Selenoproteins

### 2.1. Metal-Binding Properties of Selenoprotein P (SELENOP)

#### 2.1.1. Structural Characteristics of SELENOP

Selenoproteins contain the 21st proteinogenic amino acid selenocysteine (Sec). The human selenoproteome includes 25 selenoproteins [5]. One of these selenoproteins is selenoprotein P, also known as SELENOP, SelP, SePP, SePP1, or SeP. SELENOP is the selenoprotein that became notorious because in humans it is the only one that contains 10 Sec residues, besides 17 cysteine (Cys) and 23 histidine (His) residues [6,7]. In mammals, SELENOP is the main selenium (Se) transporter [8]. SELENOP has enzymatic and selenium transport roles and is also implicated in maintaining tissue Se hierarchy [9]. At the same time, this blood selenoprotein is a secreted heparin-binding glycoprotein, especially by hepatocytes [10,11,12], but also found in other tissues, mainly in the brain [13] and testes [14]. Other cells where SELENOP is present are the adipocytes [15] and pancreatic β-cells [16]. In addition, it is abundant in cerebrospinal fluid (CSF) where it exerts a central role in selenium homeostasis in the brain [17].

**Figure 1:**
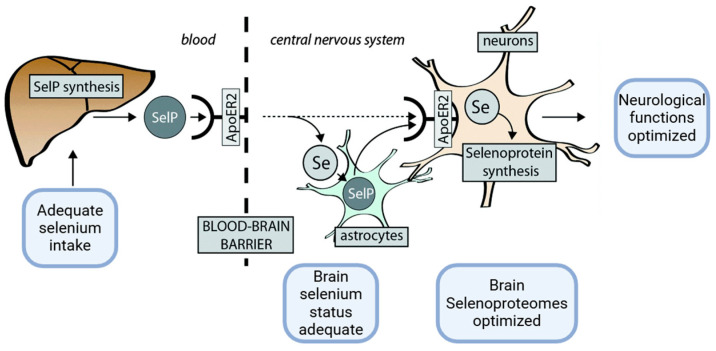
SelP is produced in the liver using selenium derived from dietary intake. At the BBB, SelP delivers selenium to the central nervous system (CNS) through the ApoER2 receptor. In the CNS, selenium is either integrated into newly synthesized SelP within astrocytes or directly transported to neurons (the dashed arrow). Neurons also acquire SelP via ApoER2 on their membranes, enabling the production of other essential selenoproteins critical for neurological function. Adapted from [21]. Created in https://BioRender.com (accessed on 3 December 2024).

Regarding the SELENOP structure, its mRNA contains ten UGA codons in the open reading frame and two SECIS (selenocysteine insertion sequence) in the 3′UTR, so its mRNA differs from other selenoproteins that contain only one SECIS element [18]. The N-terminal Sec residue of SELENOP represents an enzymatic active site for reducing lipid hydroperoxides. The other nine Sec residues of SELENOP are placed at the C-terminal region and accomplish the Se transport function. SELENOP is cleaved in limited proteolysis by plasma kallikrein between Arg235–Gln236 and Arg242–Asp243 generating the N-terminal region, which includes the 1–235 amino acid sequence involved in enzyme activity and the C-terminal region with the 243–361 amino acid sequence, which assures Se transport [19]. The N-terminal fragment has a U(Sec)XXC structural motif similar to the CXXC motif of the active site of thioredoxin that is suggestive of the reactivity of SELENOP against the protein thiols. Studies using KO SELENOP mice reported an important interaction between the C-terminal fragment and the YWTD β-propeller domain of the SELENOP receptor ApoER2, as an essential one for maintaining the selenium levels in the brain and testes [11,14,20] (Figure 1).

Miller et al. and Rueli et al. previously reported that SELENOP expression significantly increases in the brains of patients with AD [22,23]. In particular, studies of the famous His-rich domain of SELENOP are challenging in physiological and pathological conditions because besides its Sec and Cys residues, the His-rich domain is involved in the metal-binding capacity of numerous ions, such as zinc, copper, aluminum, and iron [24].

**Figure 2:**
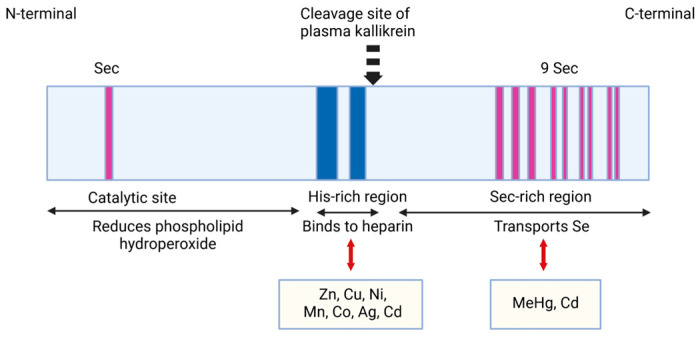
Selenoproteins P—schematic structure. The figure shows a schematic structure of SELENOP and the possible interactions between metals and each region. Adapted from [25,26]. Created in https://BioRender.com (accessed on 3 December 2024).

SELENOP contains the His-rich region in the middle of its structure with a typical heparin-binding motif XBBXB (where B is a basic amino acid) [25]. Human SELENOP has two His-rich domains in the His-rich region [9] that may explain its capacity to bind to transition metals, such as copper (Cu^2+^) and iron (Fe^2+^) (Figure 2).

These metals are known to be involved in neurodegenerative pathology, including AD, and some studies have indicated that SELENOP has neuroprotective roles due to its metal-binding properties [27,28]. At the same time, besides the His-rich domain, the redox motifs UXXC and CXXC of SELENOP are known as bivalent cation binders (for example, Cu^2+^ and Zn^2+^) [29]. The two histidine-rich domains are probably involved in heparin binding, a characteristic feature of SELENOP [26,30]. Using metal ion affinity chromatography, Sidenius et al. found the metals that can bind to SELENOP in human plasma. These metals are Cu^2+^, Ni^2+^, Zn^2+^, Co^2+^, and Cd^2+^. Co^2+^ binds most strongly to SELENOP [31].

#### 2.1.2. Metal-Binding Properties and Effects of SELENOP in Physiological Versus Pathological Neurodegenerative Conditions

In the brain, transition metal ions, such as iron, zinc, and copper, sustain many cellular processes, for example, regulating the activity of synapses, mitochondrial activity, neurotransmission, learning, memory, and the biological functions of several metalloproteins, enzymes like Cu/Zn superoxide dismutase and cytochrome c oxidase. The concentrations of these transition metals must be strictly regulated when crossing through the blood–brain barrier (BBB), so their homeostasis is accomplished by many mechanisms and factors, such as enzymes, transporters, and chaperones, all for the fine control of metal uptake and delivery to specific brain regions for preventing the random, passive transfer of the metals from circulation to the brain [32]. In the case of transition metals dyshomeostasis, the failure of the regulatory mechanisms leads to severe pathological conditions or disorders, among which are neurodegenerative diseases [33,34,35,36,37]. For example, iron, a transition metal, has recently been recognized as a putative factor in the pathogenesis of AD. At the same time, studies have reported excessive iron ions that are accumulated in the substantia nigra of patients with PD [38,39,40]. In AD, copper and zinc imbalances are associated with amyloid-β and tau pathology and influence cognitive function. In PD, iron and manganese level imbalances lead to oxidative damage followed by neuronal loss. On the other hand, toxic metals, such as lead and cadmium, cause the impairment of synaptic transmission and aggravate neuroinflammation. In AD, aluminum is involved in neurofibrillary tangle formation [41].

Amyloid-β (Aβ), α-synuclein (α-syn), and prion protein (PrP), hallmark proteins for neurodegenerative diseases, also bind some transition metals, and metal binding interferes in the aggregation process of Aβ and α-syn. Copper and iron are involved in reactive oxygen species (ROS) generation in Alzheimer’s and Parkinson’s disease patients. The Fenton reactions Cu^+^ + H_2_O_2_ →Cu^2+^ + OH^•^ + OH^−^ or Fe^2+^ + H_2_O_2_ →Fe^3+^ + OH^•^ + OH^−^ generate hydroxyl radicals (OH^•^), a highly reactive oxygen species, which, in turn, promote oxygen stress in substantia nigra pars compacta dopamine neurons by lipid membrane peroxidation, DNA damage, and protein oxidation or misfolding. Regarding PrP, studies suggested that PrP–copper interactions might modulate protection against oxidative stress, mediated by Cu^2+^, copper transport, and copper-dependent cellular signaling [42].

Se and Zn^2+^ are essential ions for proper brain function. According to the US National Institutes of Health (NIH), the total amount of zinc in the body is approximately 1.5 g in women and 2.5 g in men, mostly stored in skeletal muscle and bone. The zinc levels in serum or plasma are 80–120 μg/dL (12–18 μmol/L). Serum zinc levels below 70 μg/dL in women and 74 μg/dL in men indicate inadequate zinc homeostasis. Regarding plasma selenium, its levels are approximately 12.7 μg/dL. Selenium deficiency causes impaired cognitive and motor function [43,44]. Zn^2+^ deficiency is correlated with decreased nerve conductibility and cognitive impairment performance [45]. In AD, brain Zn^2+^ is increased [46] and can stimulate protein tau phosphorylation [47,48] which promotes the formation of neurofibrillary tangles that are characteristic of this disease [49].

**Figure 3:**
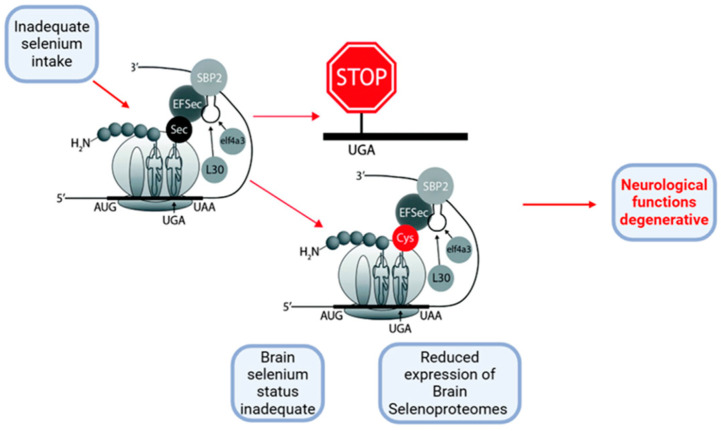
When selenium intake is insufficient, tRNA[Ser]Sec fails to decode the UGA codon as selenocysteine (Sec) and instead interprets it as a codon stop, leading to premature termination of protein synthesis. Additionally, selenocysteine may be incorrectly replaced by cysteine (Cys). Adapted from [21]. Created in https://BioRender.com (accessed on 3 December 2024).

It is well known that SELENOP is the most researched selenoprotein in AD because it has high expression and many different roles in the brain, and is associated with the expression levels of other selenoproteins [5]. For example, loss of SELENOP causes a decrease in brain expression levels of GPx4, SELENOK, SELENOM, and SELENOW [50] (Figure 3).

Zinc is the brain’s most abundant trace metal and an important element in the testes. It is essential for brain neurodevelopment and spermatogenesis, like selenium and its associated protein, SELENOP [51,52]. In the brain, zinc plays both neurotransmitter and neuromodulator roles; it is released into the synaptic terminals and modulates NMDA (N-methyl-D-aspartate) and AMPA (α-amino-3-hydroxy-5-methyl-4-isoxazole-propionate) receptors for regulating the activity of glutamatergic synapses [53,54,55,56,57].

Neurodegenerative diseases form a group of disorders with common features and etiopathogenic mechanisms. These diseases, like AD, PD, and transmissible spongiform encephalopathies (prionic diseases) are characterized by an abnormal accumulation of proteins and a specific neuronal degenerative process. Insoluble and fibrous amyloid proteins, characterized by a β-pleated sheet secondary structure and typically being partially unfolded and misfolded, tend to aggregate and accumulate as deposits in the brain. Studies have demonstrated that transition metal ions like Cu^2+^, Zn^2+^, and Fe^2+^ are strongly implicated in developing neurodegenerative disorders. Their binding may cause important conformational changes in those protein structures [28].

AD is one of these neurodegenerative disorders, it affects one in five people over 85 years of age and is fatal. This disease is characterized by two essential brain tissue pathologies, represented by abnormal protein deposits, amyloid-β (Aβ) peptide that forms the senile plaques and the neuropeptide tangles (NFT) that are bundles of paired helical filaments (PHF) whose main constituent is tau protein. The protein tau is highly soluble, and natively unfolded. It binds and promotes the assembly of microtubules. This tau protein accumulates in hyperphosphorylated NFT that can be visualized within dystrophic neuritis and cell bodies [58]. Moreover, there is a direct proportionality between the amount of tau protein damage with progressive neuronal impairment, synaptic loss, and overall decline in humans and transgenic mouse models [59,60,61].

Cu, Zn, and Fe ions bind to His residues of Aβ, trigger Aβ aggregation followed by intracellular ROS production, and finally induce cell death and cognitive decline [62]. If the tau protein is normal, it binds and stabilizes the microtubules in neurons; if it is hyperphosphorylated it aggregates in the AD-affected cells [63]. Extracellular metalloproteinases, neprilysin, insulin-degrading enzyme (IDE), and matrix metalloproteinases are involved in Aβ degradation [64]. Recent studies have demonstrated that Cu ions may be essential in tau’s physiological role. It is known that the imbalance of the transition metal ions in AD can lead to a local increase in Cu concentration and those Cu ions are then implicated in the redox reactions occurring in the neurofibrillary tangles (NFT) [65].

Some researchers have found that the tau protein or its fragments in the brain contain copper ions. Du et al. have shown that the tau fragment binds for both Cu^+^ and Cu^2+^ and that copper ions stimulate the aggregation of the tau-R2 sequence in vitro with an enhanced effect on the toxicity of tau aggregates in living neurons [66]. SELENOP could inhibit this effect by its His-rich domain. The gene for SELENOP, the SEPP1 gene, encodes two His-rich domains, one at residues 204–217 and the other at 244–250, on the protein surface [66]. The tau-R2 sequence is the second repeat sequence of the microtubule domain (MTBD), it is localized in the dense core of Alzheimer-paired helical filaments (PHF) and is essential for the biochemical properties of full-length tau protein [67,68]. In addition, Okuyama et al. found that the R2 repeat sequence has a synergistic effect on the aggregation of tau protein, and R2 included; R2 deleted tau protein isoforms have important differences in the microtubule-binding ability and PHF formation tendency [69].

Du et al. demonstrated that the SELENOP His-rich domain attenuates ROS and neurotoxicity induced by Cu^2+^/Cu^+^-tau-R2 aggregates when Cu^2+^ binds to Aβ. They measured the viabilities (by CCK-8, Cell Counting Kit-8 assay) and the ROS concentrations (with the fluorescent DCFH, 2,7-dichlorodihydrofluorescein, and quantified with flow cytometry) of mouse neuroblastoma N2A cells upon treatment with tau-R2 aggregates prepared in different conditions for 24 h. The results obtained by these researchers have shown that the SELENOP His-rich domain could accomplish good protection against tau-R2 neurotoxicity, especially by Cu^+^. At the same time, the SELENOP His-rich domain could successfully restore the neurotoxicity of Aβ induced by either Cu^+^ or Cu^2+^. So, the SELENOP His-rich domain can modulate the aggregation and neurotoxicity, caused by copper ions, of these two AD-related peptides [66]. The research also suggested that ROS production may be essential in promoting neurotoxicity induced by tau aggregates, similar to Aβ [66]. The intact tau protein aggregation in vitro depends on heparin’s presence [70]. In vivo, heparin was also found in the AD NFT. Moreover, Hondal et al. have shown that the rat SELENOP His-rich domain binds heparin under some conditions [30].

The SELENOP His-rich domain, which has a greater affinity for both Cu^+^ and Cu^2+^ ions than tau-R2, protects the primary neurons against Cu^+^/Cu^2+^-tau-R2 aggregates and modulates the Cu^+^/Cu^2+^-induced tau-R2 aggregation [66]. Using a Thioflavin S (ThS)-based assay and heparin to initiate the aggregation of tau-R2, the researchers demonstrated that the SELENOP His-rich domain almost suppressed the copper ions, presenting aggregation patterns similar to those when tau-R2 is aggregated in the absence of copper ions. In addition, when present, the SELENOP His-rich domain can disaggregate the already-formed metal–tau-R2 aggregates [66]. It is worth mentioning that certain studies have demonstrated that SELENOP interacts with the C-terminal domain of human α-tubulin [71] which is involved in the regulation of microtubule assembly, interaction with tau protein, Ca^2+^, and polyamine [72].

The metal-binding capacities of SELENOP are already well-known. Amyloid-β42 (Aβ42) also has a high affinity for metals and can bind redox-active metals like Cu^2+^ [73,74,75], Fe^2+^ [73], and Zn^2+^ [73,76,77]. In addition, SELENOP also binds to toxic metals such as Cd^2+^ [76,77] and Hg^2+^ [78]. Furthermore, the SELENOP expression in cerebrospinal fluid in patients with AD is slightly increased, Zn^2+^ concentrations are heterogenous and Zn^2+^ promotes protein aggregation [79,80]. Du et al. have shown that Aβ aggregation is inhibited, and the neuropathy is ameliorated by SELENOP binding to Zn^2+^ [29].

Studies have shown that in the case of SELENOP KO in mice brains, Zn^2+^ concentration in the hippocampus was increased and may suggest the direct binding of Zn to the His-rich domain of SELENOP [81]. In the brains of patients with AD, the compartmentalization of Cu^2+^, Zn^2+^, and Fe^2+^ is impaired and causes neuronal degeneration although the total amount of these metals remains unchanged [82]. The SELENOP receptor is ApoER2 and is known, in the brain, as ApoE, as the result of the translation of the ApoE gene [83]. One of the risk factors for AD is known to be exactly the polymorphism in ApoE [84]. Moreover, Zn^2+^ cleaves the ApoE length, making it smaller than its normal full length, and suppresses, as mentioned above, Aβ aggregation, so all these facts aggravate the disease [85]. Considering this context, SELENOP may suppress this disease by maintaining the full length of ApoE.

Studies performed by Kiyohara et al. have shown that deleting SELENOP increased the free (able to chelate) intracellular Zn^2+^, but the release of synaptic Zn^2+^ was impaired. The researchers also found that selenium deficiency could induce the release of Zn^2+^ from stores, although the Zn^2+^ storage protein metallothionein-3 (MT3) was elevated, and Zn^2+^ transporters, ZnT1 and ZnT2, were unchanged. These results may be caused by a decrease in the GPx4 selenoprotein and a subsequent increase in lipid peroxidation [81]. Regarding GPx4, Cardoso et al. suggested that it prevents neurodegeneration through ferroptosis [86]. The inhibition of GPx4 cancels ferroptosis triggering in some cancer cell lines, so the cells proliferate indefinitely [87,88]. Kiyohara et al. also demonstrated an increase in site-specific phosphorylation of tau protein in the absence of SELENOP, suggesting its role in regulating the storage of intracellular Zn^2+^ and possibly in preventing tau hyperphosphorylation in AD [81]. When SELENOP is absent, the oxidative stress in the brain increases, leading Zn^2+^ to release from MT3 [89]. Kiyohara et al. observed an incongruous impairment of Zn^2+^ release, despite an increase in the total intracellular amount of this ion that can be chelated [81]. This fact surprised the researchers because this ion is known to be localized to synaptic vesicles [90].

**Figure 4:**
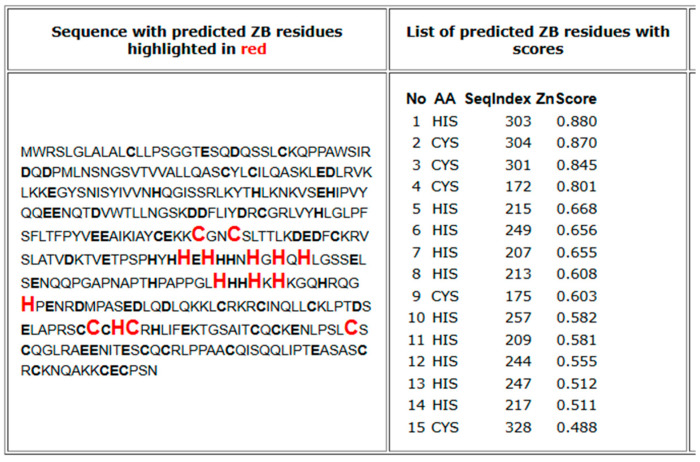
Sequence and predicted zinc-binding sites. Predicted zinc-binding (ZB) residues are highlighted in red and with larger font size (C = cysteine, H = histidine) [91].

The metal-binding domain of SELENOP can bind Zn^2+^ (with a high affinity), Co^2+^, and Ni^2+^. Utilizing the web-based tool Predzinc (https://predzinc.bioshu.se/pred/; accessed on 30 January 2025), which predicts zinc-binding proteins and sites from provided sequences, we examined the coding sequence of human SELENOP for possible zinc-binding sites. Our analysis indicated that several histidine and cysteine residues within the His-rich metal-binding domain are likely to serve as Zn^2+^-binding motifs (Figure 4).

Kiyohara’s research demonstrated that selenium deficiency/absence, inhibition of GPx4, and loss of antioxidant function in SELENOP KO mice are responsible for the increased Zn^2+^ levels. The reason for the presence of the metal-binding domain of SELENOP remains unknown. Researchers believe that it is possible that the affinity of SELENOP for the ApoER2 receptor could be altered when Zn^2+^ is binding, allowing the regulation of selenium by excess Zn2+. They concluded that SELENOP may have an essential role in the maintenance of the brain Zn^2+^. SELENOP gene translation can be related to Zn^2+^ metabolism and synaptic release, so SELENOP is probably implicated in neuronal and synaptic functions [81]. In AD, Zn^2+^ is increased, interacts with amyloid-β42 (Aβ42), and can promote protein tau phosphorylation [46,48]. Thus, in AD, by binding Zn^2+^, SELENOP can associate with beta plaques [72].

#### 2.1.3. The Properties and Roles of SELENOP in Binding Toxic HEAVY Metals

Many studies were performed regarding the interaction between selenium, SELENOP, and toxic environmental pollutants such as methylmercury, As (arsenic), and Cd^2+^ [92,93]. Cd^2+^ binds to proteins, especially to metallothionein and SELENOP. Cd^2+^ binds to metallothionein by CXXC or CXC motif [94], and SELENOP by CXU, UXC, or UXU motif in its C-terminal domain, Sec-rich. Sasakura et al. purified SELENOP fractions from male Wistar rats’ plasma. The researchers studied, using the HPLC-inductively coupled argon plasma-mass spectrometry method (ICP MS), the interaction between transition metals, such as Ag^2+^, Cd^2+^, Hg^2+^, and selenium (Se), in the bloodstream. In vitro experiments have shown that transition metal ions and selenide generated from selenite in the presence of glutathione (GSH) or sulfide (Na_2_S), form a (metal–Se/S) complex. This complex subsequently binds to SELENOP, forming a ternary complex (metal–Se/S)–SELENOP in the bloodstream. Cd^2+^ and Ag^2+^ react with the reduced form of Se to form the (Cd-Se) and (Ag-Se) complex at first, then this complex to SELENOP in the bloodstream. The (metal–Se)–SELENOP complex was formed with Cd^2+^ and Ag^2+^ with the same efficiency as with Hg^2+^ [95]. In addition, computer models have suggested the binding of Cd^2+^ to His-rich plasma glycoproteins [96,97].

Studies have demonstrated that Cd^2+^ induces oxidative stress while selenium reduces toxicity, especially in kidneys and testes. When low concentrations of Cd^2+^ in mice are administrated, lipid peroxide accumulates in the testes, and ferroptosis is induced [98]. Toyama et al. research concluded that SELENOP KO in mice testes almost annihilated GPx4 expression, an enzyme involved in the reducing reaction of phospholipid hydroperoxide [25]. SELENOP expression decreases after exposure of the HepG2 hepatocarcinoma cell line to high doses of Cd^2+^ [99]. When enough selenium amount is provided, SELENOP expression is optimal and able to protect against Cd^2+^ toxicity, but when Cd^2+^ is excessively increased, SELENOP expression in the liver decreases causing oxidative stress, and triggering ferroptosis [25].

The Nunavik Inuit adults are one of the groups exposed to high concentrations of methylmercury and selenium because of the daily consumption of seafood and marine mammals. Achouba et al. analyzed the Inuit plasma using LC-ICP/MS and found the highest mercury concentration at the same fraction of SELENOP [100]. Chen et al. studied the mercury miners in China who were occupationally exposed to inorganic mercury, and found that both SELENOP and GPx3 were bound, in serum, to mercury [101]. SELENOP is an essential methylmercury-binding selenoprotein in plasma, indicating its role in detoxifying from heavy metals.

In a rat model created by Liu et al., SELENOP was the main target of methylmercury in the plasma. Methylmercury was administered at 4 mg/kg for 4 weeks, within methylmercury-containing water. The researchers concluded that 73% of mercury in plasma is bound with SELENOP, the major mercury form of transportation, despite the abundance of serum albumin [102]. In rats, SELENOP levels in plasma decreased by methylmercury intoxication for 4 weeks of exposure to 20 ppm, which was not the case with lead (Pb) and cadmium (Cd) exposures [103].

Other experiments have demonstrated that simultaneous and equimolecular administration of inorganic mercury (HgCl_2_) and selenite prevented toxicity. This fact made researchers suggest the formation of an equimolar (HgSe)n, bound to a specific plasma protein in plasma. Later, this specific protein proved to be SELENOP [104]. The researchers found that inorganic mercury or selenite failed to bind to SELENOP, so the (HgSe)n complex interacted ionically with SELENOP, but not Hg^2+^. They estimated the number of HgSe complexes bound to SELENOP to be approximately 100, and they suggested at least 35 binding sites on SELENOP [105]. The effects of methylmercury and inorganic mercury on the selenium transporting function in SELENOP have not been studied until now, so future experiments on this theme will be needed.

Free inorganic selenium may be protective against the toxicity of various metals, such as Pb, Hg, As, and Cd. It inhibits their toxicity by directly binding to some metals. At the same time, selenium represents a source of selenoprotein synthesis and protects cells by scavenging ROS [106]. In addition, other researchers found that selenium derivatives prevent oxidative damage through binding to Cu^2+^ and Fe^2+^. Sec directly inhibited the “in vitro” DNA damage induced by Cu^2+^ and Fe^2+^, despite a low GPx activity, suggesting that the first mechanism is represented by binding the metal to selenium [107].

Studies in humans regarding arsenic (As) have shown that selenium may reduce As accumulation overall in the organism and protect the skin lesions produced by As [108]. Another study demonstrated that dietary selenium blocked the evolving cancer effects caused by As on UV radiation-induced carcinogenesis in mouse skin. It is thought that selenium probably prevented As retention in mouse skin by an As-Se metabolite, a seleno-bis (S-gluthationyl) arsenic ion, found in traces, in the mouse’s liver. Moreover, the formation of this compound was more likely to accomplish the As-blocking effect by Se than a mechanism involving oxidative stress reduction [109]. Naderi et al. emphasize the importance of recognizing that, although selenium offers protective and ameliorative effects against metal toxicity, its interaction with toxic elements may prolong their persistence in tissues, potentially resulting in toxic effects [110].

Yue et al. have demonstrated that the overexpression of SELENOP, usually noted as SELENOP-H, with its His-rich motif (known that does not contain Sec residues), suppressed the metal-induced aggregation and neurotoxicity of both Aβ and the tau protein in vitro in triple transgenic AD (3 × Tg-AD) mice. SELENOP-H can accomplish these actions by regulating tropomyosin receptor kinase B (TrkB) signal transduction and Zn^2+^ homeostasis. The SELENOP-H gene, packaged in rAAV9 (a human-derived adeno-associated virus vector for efficient transgene expression in mouse cingulate cortex), was administered into the hippocampal CA3 regions of mice by stereotaxic injections. After four months, the researchers ascertained improved spatial learning and memory deficits, reduced neuron damage, reduced synaptic protein loss, the inhibition of both tau protein pathology and Aβ aggregation, the activation of brain-derived neurotrophic factor, BDNF, and the family of proto-oncogenic tyrosine kinases genes, Src-mediated TrkB signaling, and increased MT3 and ZnT3 levels, so SELENOP-H restored Zn^2+^ homeostasis in the mice model of AD [111].

In 2020, Solovyev et al. commented that the clearest evidence regarding the link between SELENOP and AD is due to in vitro studies, so more animal models and studies on humans are needed for solid reliable conclusions. In addition, the interaction between SELENOP and its ApoER2 receptor regarding their effects on synaptic signal transmission needs more deepening studies to certify their precise involvement in synaptic dysfunction in AD [112].

In the first “in vivo” prospective cohort study, Vinceti et al. suggest that concentrations of two biomarkers of full-length SELENOP levels, in serum and CSF (cerebrospinal fluid), can predict the conversion towards dementia among subjects with MCI (mild cognitive impairment). Thus, high levels of SELENOP seem to exert beneficial and toxic effects depending on its dose and might have deleterious effects on cognitive function; caution is needed regarding this conclusion due to study limitations (limited sample size, lack of post-mortem neuropathological examinations). Regarding dementia types, the researchers indicate that SELENOP can predict dementia conversion from MCI more pronounced for frontotemporal dementia and Lewy bodies dementia rather than Alzheimer’s dementia, probably because of their different pathological processes and different risk factors that induce those diseases. It may be understandable that the detrimental effect of too high levels of SELENOP could trigger more mechanisms of non-Alzheimer’s dementia than forming the β-amyloid plaques and neurofibrillary tangles deposition which are characteristic of Alzheimer’s disease. Vinceti et al. studies also suggest that when assessing the relation between SELENOP levels and the incidence of non-Alzheimer’s dementia it is necessary to rely on CSF concentrations rather than its serum levels, differently from what appears to be true in Alzheimer’s dementia. Further “in vivo” human studies are still needed regarding the relation between brain SELENOP and dementia risk [113].

### 2.2. Metal-Binding Properties of Selenoprotein W (SELENOW)

In the brain, SELENOW is one of the selenoproteins whose expression is most affected by SELENOP [50], although dietary selenium deficiency does not decrease SELENOW levels, it reduces GPx activity [114].

SELENOW, like other selenoproteins, is also a GSH (glutathione)-dependent antioxidant and is involved in redox reactions [114]. SELENOW is highly expressed in the cerebral cortex, dentate gyrus, and hippocampus of postpartum rats, and the brain and spinal cord of developing embryos [115,116]. SELENOW has a functional role and is expressed in synapses and decreases in mice’s SELENOP knock-out [117]. Chen et al. concluded that Cys37 of SELENOW forms a disulfide bond with Cys322 of tau protein to inhibit tau protein aggregation, suggesting that, besides SELENOP, SELENOW may also be involved in AD. However, further research is still needed to clarify this process in vivo [118].

### 2.3. Metal-Binding Properties of Selenoprotein M (SELENOM)

SELENOM is another selenoprotein expressed in various tissues, but highly expressed in the brain [119]. Studies performed in mouse neurons have shown a protective role of SELENOM against ROS in the brain, and also a role in calcium regulation, suggesting the potential implication of SELENOM in AD [120].

Recently, Du X et al. demonstrated that SELENOM reduced the intracellular ROS levels and inhibited Aβ aggregation in HEK293T cells, containing the CXXU motif [29,121]. It is important to mention that the CXXU (or UXXC, CXU, and UXC) redox-active motif is also present in other selenoproteins, such as SELENOW, SELENOH, and SELENOT, in addition to SELENOM and SELENOP [122]. Moreover, studies have highlighted that the CXXC-like motif can bind Zn^2+^ and Cu^+^ in proteins. Sec and Cys share many similarities, and selenium and sulfur are in the same group of elements in the periodic table, and share some properties, so it is understandable that SELENOM, as well as the other selenoproteins containing the CXXU motif, may act as metal regulators, so they may also modulate metal-induced Aβ aggregation and neurotoxicity in AD.

Regarding these facts, Du et al. have cloned and expressed the His-rich domain of SELENOP, named SELENOP-H (residues 188–263), and a mutant SELENOM (U48C, named SELENOM’). They studied the metal-binding properties of SELENOP-H and SELENOM’ and their abilities to regulate Zn^2+^-induced Aβ aggregation and neurotoxicity [29]. Du et al. used in their experiments a Cys homolog, to investigate the properties of the native protein form, because it was difficult to prepare selenoproteins in a heterologous expression system. The researchers concluded that the secondary structure of SELENOM’ was changed by binding metals, so Zn^2+^ and Cd^2+^ binding increased the α-helix content but decreased the β-sheet content. SELENOM’ chelated Zn^2+^/Cd^2+^ at the CXXC (or CXXU, in a presumed manner, in the wild-type protein) motif. SELENOW, SELENOH, and SELENOT could also have metal-binding properties [29].

Regarding SELENOP-H Zn^2+^/Cd^2+^ binding, the research has shown an increase of absorption at 250 and 280 nm, suggestive of the ligand-to-metal charge transfer band process, the dominance of α-helix content, and an increase in the β-sheet content. SELENOM’ exhibited a small effect on Aβ42 fibrillization, while SELENOP-H accelerated the fibrillization rate, but decreased the amount of the fibrils formed [29]. In addition, similar studies, in consensus with Du et al. experiments, reported that proline-rich whey peptides suppressed Aβ42 fibrillization [123]. Furthermore, proline-rich peptides had neuroprotective effects and prevented the neurodegeneration caused by Aβ25–35 in the hippocampus [124,125]. The fact that SELENOP is co-localized with Aβ plaques in the postmortem tissue from patients with hallmark lesions of AD, suggests that SELENOP may interact directly with Aβ for neuroprotection [72].

Regarding Aβ42 aggregation, Du et al. demonstrated that both SELENOM’ and SELENOP-H inhibited Aβ42 aggregation induced by Zn^2+^ ions, but SELENOM’ exerted a higher inhibitory effect compared to SELENOP-H, probably due to the higher affinity of SELENOM’ for Zn^2+^. At the same time, both these selenoproteins also induced the formation of some fibrils. The researchers concluded that SELENOM’ and SELENOP-H can modulate the neurotoxicity of Aβ42 and protect neuronal cells against Aβ42-Zn^2+^ toxicity [29].

In the brain, other important effects of metal ions’ actions, including zinc, copper, and iron, are the formation of ROS and the promotion of oxidative stress [126,127]. In the experiments performed by Du et al., ROS production in N2A cells upon treatment with Aβ42 fibrils or Zn^2+^-Aβ42 non-fibrillar aggregates, in the presence of either SELENOP-H or SELENOM’, was detected with the fluorescence DCFH (2,7-dichlorodihydrofluorescein) and quantified by flow cytometry [29]. Many studies have revealed that ROS production generated by Aβ42 and oxidative damage of cerebral endothelial cells and astrocytes are mainly caused by activation of the NADPH oxidase, which is known to promote superoxide formation [128,129,130]. SELENOP-H and SELENOM’ suppressed the ROS production generated by Zn^2+^-A β42 aggregates [29].

Hwang et al. have demonstrated that the transcription of SELENOM is inhibited in a significant manner in the brains of familial AD transgenic mice overexpressing a human mutant presenilin 2 (PS2) gene, which disrupts Ca^2+^ homeostasis by facilitating the transfer of Ca^2+^ from the endoplasmic reticulum to mitochondria [131].

### 2.4. Metal-Binding Properties of Selenoprotein S (SELENOS)

SELENOS is a selenoprotein also involved in AD. Experiments have revealed that suppression of ER-stress-induced SELENOS expression increases protein tau phosphorylation and phosphorylated tau aggregation [132]. These findings contradict studies demonstrating that SELENOS is involved in Aβ synthesis, hyperphosphorylation, and tau protein aggregation [133].

Parkinson’s disease is also a progressive neurodegenerative disorder characterized by losing dopaminergic neurons and the formation of Lewy bodies in substantia nigra pars compacta. The consequence is dopamine deficiency which causes motor manifestations like rigidity, tremor, and bradykinesia. It also causes non-motor manifestations that include sleep disturbance and constipation. The prevalence of PD is 1–2% at the age of 60 years and 3–8% between 85 and 89 years of age [134]. The incidence is higher among men than women.

Most PD cases are sporadic, and are determined by diverse etiologies, including environmental factors and genetic causes. Dopaminergic neuronal degeneration can be triggered by excessive exposure to toxic metals, such as Hg, Pb, Cu, Zn, Fe, Mn, Al, As, Cd, and Se, which enter the brain via the BBB. In addition, the pathogenesis is completed by oxidative stress, mitochondrial dysfunction, environmental toxins, autophagy, misfolding and abnormal protein accumulation of α-syn protein, and neuroinflammation [135]. Regarding long-term heavy metal exposure, neuroinflammation is produced by increased pro-inflammatory cytokines (IL-1β, IL-6, TNFα), and leads to neuronal loss [136].

Selenium may interact with α-syn contained in Lewy bodies, leading to protein aggregation and crosslinking [137]. α-Synuclein is a 140-amino-acid copper-binding protein that contributes to the formation of Lewy bodies, the hallmark of PD. It has three distinct domains: the first is the N-terminal domain (residues 1–60), containing two anchoring sites for Cu^2+^ ion at Met1(NH_2_) and His50 (Im), followed by the so-called non-amyloidogenic domain (residues 61–95), and the third is the negatively charged C-terminal domain residues 94–140), rich in Asp and Glu residues, which represents potential additional metal-binding sites [28]. Many studies have revealed that increased Se deposits in substantia nigra in PD patients cause oxidative stress and damage to the nigrostriatal dopaminergic neurons [12]. In contrast, Se is considered an antioxidant since it is incorporated into selenoproteins, such as SELENOS and SELENOP, and is also involved in PD [138].

SELENOS is localized in the membrane of the endoplasmic reticulum (ER). Its roles are to process and transport misfolded proteins out of the ER into the cytoplasm, where proteasome degraded them, after polyubiquitination. SELENOS is induced by ER stress. Incomplete folded proteins are either left in the ER to complete folding or are targeted for ubiquitination and destructed [139]. The researchers concluded that SELENOS might prevent misfolded α-syn transportation through the UPR (unfolded protein response) pathway and determine the decrease of its aggregation [140].

SELENOS is physiologically highly expressed in the brain and is involved in neurons in the modulation of ER homeostasis [141]. SELENOS reduces ER stress and the inflammation process by lowering pro-inflammatory cytokines. The Salaramoli et al. experiment has indicated the same results in PD and AD referring to SELENOS levels, which were associated with UPDRS score and α-syn levels. As well as SELENOS decreased tau protein aggregation in AD, it reduced α-syn aggregation in PD serum [140].

Another study concluded that co-localization and concentration together of SELENOP and α-syn, at one site, suggest a possible relationship during the development of PD. At this site, SELENOP may connect with α-syn during the early stages of the disease, since α-syn is often present in presynaptic terminals of dopaminergic neurons. Aggregates of misfolded proteins may, particularly, interact with SELENOP. Another hypothesis indicates that SELENOP may be selectively linked to one or more proteins and co-localized with tau protein, which is present in some Lewy bodies, in neurofibrillary tangles [142]. Salaramoli et al. have shown that the levels of SELENOP in serum were negatively associated with α-syn aggregation. This fact leads to the conclusion that SELENOP might transport more Se to the brain and neural cells, which induces more SELENOS to prevent aggregation of misfolded proteins. These hypotheses need to be further researched. In addition, all the results suggested that a change in the serum levels of Se and SELENOP may be a sign of PD or a risk for developing the disease. SELENOS levels in PD are directly affected by changes in SELENOP levels, and SELENOS contributes to changing the levels of aggregated proteins, though its exact role in Parkinson’s disease remains unclear [140].

## 3. Conclusions

The investigation of the functions of selenoproteins in the brain, which are representative of selenium action and its physiological functions, also includes their metal-binding properties. Selenoproteins GPx4, GPx1, SELENOP, SELENOK, SELENOT, SELENOM, SELENOS, and SELENOW are highly expressed, so they are closely associated with brain function. Selenoproteins exert multiple metal-binding abilities, for example, copper, zinc, cadmium, mercury, cobalt, nickel, silver, and manganese (Table 1).

Selenoproteins have essential physiological roles and are also related to brain pathological disorders, especially neurodegenerative diseases. Existing data reveal that these selenoproteins are involved in Alzheimer’s and Parkinson’s disease because they participate in pathological protein aggregation, synaptic dysfunctions, and neuroinflammation. While there is substantial evidence of the antioxidant and redox-regulating functions of selenoproteins, their metal-binding properties remain insufficiently characterized. Few studies have explored the molecular mechanisms by which these proteins interact with essential or toxic metals, such as zinc, copper, or iron, which are implicated in neurodegenerative diseases.

The brain’s susceptibility to metal dysregulation, as seen in Alzheimer’s disease (amyloid plaques enriched with iron and copper) or Parkinson’s disease (α-synuclein aggregation involving iron), suggests an urgent need to understand how selenoproteins contribute to maintaining metal homeostasis and mitigating neurotoxicity.

Structural studies on metal-binding domains in selenoproteins are sparse. Understanding how selenium’s incorporation influences the binding affinity and selectivity of selenoproteins for metals is critical for elucidating their functional roles in neurodegeneration.

Directions for future research could include elucidating metal-binding mechanisms (advanced techniques such as X-ray crystallography, NMR spectroscopy, and molecular dynamics simulations might resolve structural details); testing the effects of selenium supplementation or selenoprotein mimetics on metal dysregulation in neurodegeneration or developing small molecules or peptides that mimic selenoprotein activity, particularly for chelating neurotoxic metals or enhancing antioxidant defense; extend research to lesser-known selenoproteins, such as SELENOM or SELENOF, which might play roles in intracellular metal trafficking or storage, based on their cellular localization and function. Regarding Se supplementation in AD and MCI (mild cognitive impairment), studies observed improvement in Se levels, GPx (glutathione peroxidase activity), and some cognitive tests in MCI patients and mini-mental scores in AD patients. Even though Se supplementation represents a good alternative for alleviating patients’ status and symptoms, further investigations are required to evaluate the effects of Se on the cognitive deficit and oxidative stress associated with AD and MCI, as well as the long-term effects of Se supplementation [146].

By addressing these gaps, future research can clarify the interplay between metal-binding selenoproteins and neurodegeneration, paving the way for novel diagnostic and therapeutic strategies.

## Figures and Tables

**Table 1:** Binding patterns of selenoproteins and selenium to various metals. From [33].

		Binding Site	Metal	References
Selenoprotein P		His-rich domain	Zn, Cu, Ni, Mn, Co, Ag, Cd	[29,31,66,95]
		Sec	MeHg, Cd	[99,102]
Other selenoproteins	TrxR	Sec	Au, Pt, Pd	[143,144]
	GPx	Sec	Au	[143,145]
Selenium			Hg, Ag, Pb, As, Mn, Cu, Fe	[106,107,109]

## Data Availability

No new data were created or analyzed in this study.
